# *I*-GSEA4GWAS v2: a web server for functional analysis of SNPs in trait-associated pathways identified from genome-wide association study

**DOI:** 10.1007/s13238-014-0114-4

**Published:** 2014-11-20

**Authors:** Kunlin Zhang, Suhua Chang, Liyuan Guo, Jing Wang

**Affiliations:** Key Laboratory of Mental Health, Institute of Psychology, Chinese Academy of Sciences, Beijing, 100101 China

**Dear Editor,**

The standard data analysis of genome-wide association study (GWAS) is based on single SNP (single nucleotide polymorphism), thus it ignores combined effect of modest SNPs/genes. To solve this problem, pathway-based analysis (PBA) has been introduced to GWAS data analysis. PBA aims to identify biological functions and mechanisms associated with complex trait (Wang et al., 2007; Wang et al., 2010). By now it has been one of the key ways to interpret GWAS data (Wang et al., 2010).

The results of PBA are trait-associated pathways, which represent combined effect of modest genes. Further validation study needs to explore candidate causative SNPs from the PBA-identified pathways, by annotating the SNPs of the genes involved in pathways based on genomic features including protein coding features and non-coding features. For coding features, the SNPs impacting protein functions (such as deleterious non-synonymous sites) have been widely investigated, and the information has been well collected in some databases like Ensembl (Flicek et al., 2014). Meanwhile, to assign the biochemical function of the non-coding part of human genome (particular functional elements for gene expression regulation), the Encyclopedia of DNA Elements (ENCODE) project (Bernstein et al., 2012) has identified plenty of regulatory regions, like DNase I hypersensitive sites (DHSs) and transcription factor binding sites (TFBSs) in human genome. Particularly, the result of ENCODE indicates that most of the SNPs identified by GWASs are enriched within non-coding functional elements, with a majority residing in or near ENCODE-defined regions, including DHSs and TFBSs across several cell types (Bernstein et al., 2012). By now there have been several tools, such as GenomeRunner (Dozmorov et al., 2012) and GREAT (McLean et al., 2010), which can annotate and analyze the non-coding genomic features for input genomic regions. However, these tools are general tools and not specific for trait-associated pathways identified from GWAS. Furthermore, coding region annotation and linkage disequilibrium (LD), which is the basic concept of GWAS, need to be considered. On the other hand, except for genomic features, expression quantitative trait loci (eQTLs) analysis is widely utilized to interpret biological mechanisms of GWAS-identified variants (Cookson et al., 2009). Taken together, a PBA tool combined with solutions for functional analysis (considering all above issues) of SNPs in PBA-identified pathways associated with trait will provide an objective and comprehensive way to interpret GWAS data.

In our previous work, we have developed a PBA web server, *i*-GSEA4GWAS (*improved* gene set enrichment analysis for GWAS), which detects pathways associated with traits by applying an improved gene set enrichment analysis (*i*-GSEA) (Zhang et al., 2010). Here, we report a new version, *i*-GSEA4GWAS v2, which is featured by implementing both *i*-GSEA and follow-up functional analysis for SNPs in trait-associated pathways identified by *i*-GSEA as well as their linkage disequilibrium (LD) proxies. The functional analysis of *i*-GSEA4GWAS v2 is based on putative functional SNP annotation data from Ensembl, regulatory regions from ENCODE and eQTLs data. Both annotation analysis and enrichment analysis were conducted for each type of functional elements. Data sources used for functional analysis were shown in Tables S1–3. Details about the annotation and enrichment analysis methods are in Materials and Methods section in Supplementary Materials. Fig. 1 shows the analytical framework of *i*-GSEA4GWAS v2.

**Figure 1:**
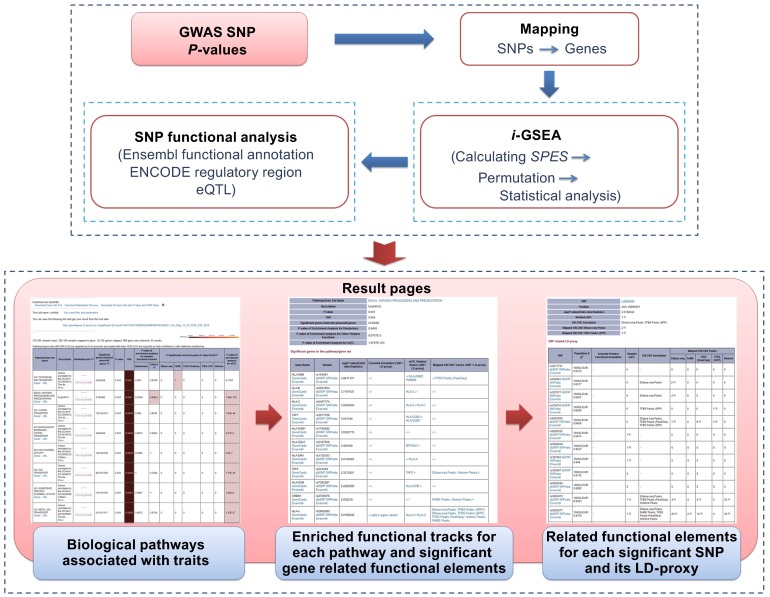
The analytical framework of *i*-GSEA4GWAS v2

*I*-GSEA4GWAS v2 is freely available at http://gsea4gwas-v2.psych.ac.cn/. It supports all major browsers. The main options of it are in Table 1. There is no any restriction to use it by academics and non-academics. It is written in Java and JSP and distributed using Apache and Tomcat web servers. *I*-GSEA4GWAS v2 was run in an upgraded web server (DELL R910 with 256G memory, 40 core CPUs and almost 10T storage) than *i*-GSEA4GWAS to improve the computing speed and work load. It is platform independent and easy to use by genetic and biological researchers; web browser is the only requirement to use it.

**Table 1:** The main options of *i*-GSEA4GWAS v2

Option	Parameter name (separated by “,”)	Default value	Description
Select mapping rules of SNPs→genes	20 kb, 5 kb, 100 kb, 500 kb, within gene, customized (0–500 kb)	20 kb	The threshold of SNP mapping to its nearest gene. Radio box
Gene set database	KEGG, BioCarta, GO biological process, GO molecular function, GO cellular component, customized	KEGG, BioCarta, GO biological process, GO molecular function, GO cellular component	The pathway/gene set database used for PBA search. Check box
Number of genes in gene set	[0, infinite]	[20, 200]	The size of gene set
Functional analysis	Yes, no	Yes	All the options below will work only this option is set to “Yes”. Radio box
Select LD data source	1000 genome population, HapMap III population	1000 genome population	LD data source (1000 Genomes or HapMap III). Radio box
1000 genome population	EUR, AMR, ASN, AFR	EUR	1000 genome population. Check box
HapMap III population	CEU, CHB, JPT, YRI, ASW, CHD, GIH, LWK, MEX, MKK, TSI	CEU	HapMap III population. Check box
Select functional data source	Ensembl putative functional variants, ENCODE regulatory feature peaks, Expression quantitative trait loci (eQTLs)	Ensembl putative functional variants, ENCODE regulatory feature peaks, expression quantitative trait loci (eQTLs)	Functional annotation data source for Ensembl. Check box
Data type	DNase-seq Peaks, FAIRE Peaks, TFBS Peaks (PeakSeq), TFBS Peaks (SPP), Histone Peaks	DNase-seq peaks, FAIRE peaks, TFBS peaks (PeakSeq), TFBS peaks (SPP), histone peaks	Data type of ENCODE regulatory feature peaks. Check box
Tissue	All, blastula, blood, bone, brain, breast, cerebellar, cervix, colon, connective, embryonic, epithelium, eye, fetal, foreskin, gingiva, gingival, heart, induced, kidney, liver, luminal, lung, mammary, monocytes, muscle, myometrium, pancreas, pancreatic, prostate, skin, spinal, testis, urothelium, uterus	All	Tissue of ENCODE regulatory feature peaks. Check box

We applied *i*-GSEA4GWAS v2 to analyze 312,565 SNP *P*-values of a schizophrenia GWAS with 871 schizophrenia cases and 863 healthy controls (all of European origin) at discovery stage (Need et al., 2009). The program maps the SNPs to the nearest genes within 20 kb upstream/downstream, searches KEGG (Kanehisa et al., 2014), BioCarta (http://www.biocarta.com/), and GO (Ashburner et al., 2000) for trait-associated pathways, and extracts linkage disequilibrium (LD) proxies for functional analysis from 1000 Genomes EUR. The result identified 19 pathways with FDR < 0.05 (Table S4). Among these 19 pathways, one (‘antigen processing and presentation’) was significantly enriched in Ensembl other putative functional sites, 8 of them were significantly enriched in at least one track of ENCODE regulatory elements and 14 of them were significantly enriched in eQTL, which indicated most SNPs in these pathways were in non-coding regions which may regulate the gene expression (Bernstein et al., 2012). For ‘antigen processing and presentation’, it is suggested that in schizophrenia, cellular mechanisms that are involved in antigen processing and presentation could be less efficient (Craddock et al., 2007). Functional analysis for the significant SNPs and their LD proxies in this pathway indicates that LD proxies (rs9260107 and rs9260118) of rs2860580 in *HLA-A*, LD proxies (rs2072895 and rs2844846) of rs1362126 in *HLA-F* and LD proxy (rs3100139) of rs2254835 in *B2M* are annotated as splice region; LD proxy (rs2072895) of rs1362126 in *HLA-F* is annotated as deleterious. The three genes are all related with major histocompatibility complex (MHC) class I molecules, which is consistent with the recent findings about the contribution of MHC region site to schizophrenia (Ripke et al., 2013). Four schizophrenia-associated pathways ‘potassium ion transport’, ‘regulation of heart contraction’, ‘voltage gated potassium channel complex’, and ‘potassium channel activity’ were enriched in ENCODE FARE track “wgEncodeOpenChromFaireMedulloPk” (a cell line of brain, as shown in Table S5), indicating many SNPs in these pathways may regulate the gene expression in brain. These functional SNPs and genes we identified may lead important function in schizophrenia and deserve further validation.

In summary, this release of *i*-GSEA4GWAS v2 adds the important features towards an objective and comprehensive GWAS data interpretation, namely functional analysis for SNPs in trait-associated pathways. The functional analysis implemented in our web server for SNPs in trait-associated pathways considers linkage disequilibrium (LD) information, three categories of features (coding, non-coding and eQTLs) and enrichment analysis, which is a very comprehensive tool/module in comparison to available web-based tools for functional analysis (Table S6). The functional analysis result would facilitate to understand all kinds of main features (coding, non-coding and eQTLs) of the pathway related SNPs and select candidate causal SNPs, which will further contribute to the interpretation of GWAS data. To our knowledge, this is the first effort that SNP functional analysis is implemented in a PBA tool for GWAS. In future research, we will continue to update *i*-GSEA4GWAS v2 with latest pathways and annotation data of genomic features.

## Electronic supplementary material

Below is the link to the electronic supplementary material.

Supplementary material 1 (PDF 98 kb)
