# Supplementary material for: I-GSEA4GWAS v2: a web server for functional analysis of SNPs in trait-associated pathways identified from genome-wide association study
Source: Protein Cell. 2014 Nov 20;6(3):221–4. doi: 10.1007/s13238-014-0114-4 (PMC4348241; doi:10.1007/s13238-014-0114-4)
Supplement: Supplementary file 1 — Supplementary material 1 (PDF 98 kb) [file 13238_2014_114_MOESM1_ESM.pdf]

Supplementary Materials

I-GSEA4GWAS v2: a web server for functional analysis of SNPs in trait-associated pathways identified from genome-wide association study

Kunlin Zhang [#], Suhua Chang [#], Liyuan Guo, Jing Wang [*]

Key Laboratory of Mental Health, Institute of Psychology, Chinese Academy of Sciences, Beijing 100101, China

[#] Equal contribution.

* To whom correspondence should be addressed. Prof. Jing Wang, 16 Lincui Rd., Chaoyang District, Beijing 100101, China. Tel: +86 10 6485 5841, Fax: +86 10 6485 5841, Email: wangjing@psych.ac.cn.

Running title: Functional analysis of SNPs in trait-associated pathways

Keywords: functional analysis, SNPs, trait-associated pathways, genome-wide association study (GWAS)

Materials and methods

General analytical framework

With the GWAS SNP *P*-values as input, the program firstly performs *i*-GSEA to identify pathways associated with trait. Then it carries out functional analysis for both the most significant SNPs of the genes involved in the pathways and their LD proxies extracted from user-defined HapMap (Altshuler et al., 2010) or 1000 Genomes (Abecasis et al., 2012) populations. The functional analysis of SNPs includes 1) annotation based on Ensembl putative function annotation, ENCODE regulatory regions and eQTLs, and 2) enrichment analysis implemented for each type of functional annotation to explore if the significant SNPs in each trait-associated pathway are significantly enriched in these functional elements. Finally, the trait-associated pathways with detailed results of SNP functional analysis are displayed. **Figure 1** shows the analytical framework of *i*-GSEA4GWAS v2.

Functional analysis

To perform functional analysis for SNPs in each trait-associated pathway, we started from the most significant SNPs of the significant genes (the genes mapped by at least one of the top 5% SNPs) (Zhang et al., 2010). We first extracted the LD proxies of the most significant SNPs (the SNPs which have $r^2 > 0.8$ with the most significant SNPs) based on the LD information of specific population(s) of HapMap phase 3 or 1000 Genomes Integrated Phase 1. Then we performed functional analysis for the most significant SNPs and their LD proxies. The functional analysis included annotation and enrichment analysis.

The first type of annotation was to annotate SNPs impacting protein function (deleterious non-synonymous or others including splice donor variant, stop lost, incomplete terminal codon variant, inframe insertion, transcript ablation, splice acceptor

variant, frameshift variant, stop gained, initiator codon variant, splice region variant or inframe deletion), which were based on the SNP annotation data in Ensembl. The statistics for each type of SNPs annotated on different function types was shown in **Supplementary Table 1**. The second type of annotation was to map SNPs to ENCODE regulatory regions. The uniform peaks for DNase (DNase-seq Peaks), FAIRE (FAIRE peaks), TFBS (TFBS Peaks (SPP) and TFBS Peaks (PeakSeq)) and Histone (Histone Peaks were downloaded from ENCODE (http://genome.ucsc.edu/ENCODE/downloads.html). The statistics of tracks for each type of ENCODE regulatory features was shown in **Supplementary Table 2**. For histone peaks, only the peak regions marked by active-associated histones, including H3K4me1, H3K4me2, H3K4me3, H3K9ac, H3K27ac, H3K36me3, H3K79me2, H4K20me1 and H3K9me1, were included as rSNPBase (Guo et al., 2014). To annotate the non-coding features of SNPs, which might regulate gene expression, the mapping of SNP to ENCODE regulatory region was established if the SNP was within the regulatory region (peak) and both the SNP and regulatory region were within the 5kb upstream of gene coordinates. The third annotation is based on eQTLs data from several databases, which contained the relationship between SNPs and their affected genes. The data sources and statistics for eQTL data was shown in **Supplementary Table 3** (Myers et al., 2007; Stranger et al., 2007; Schadt et al., 2008; Veyrieras et al., 2008; Dimas et al., 2009; Gibbs et al., 2010; Montgomery et al., 2010; Pickrell et al., 2010; Zeller et al., 2010; Innocenti et al., 2011; Gaffney et al., 2012; Xia et al., 2012; Mangravite et al., 2013).

After SNP annotation, enrichment analysis was carried out based on binomial test for each type of functional annotation to explore if the significant SNPs in each trait-associated pathway are significantly enriched in these functional elements. Briefly, for each type of functional element, we estimate an empirical *p* based on proportion of functional elements in the whole genome, then perform binomial test based on it for each

trait-associated pathway. The enrichment *P*-values are calculated for each type of functional elements respectively, including each type of ENCODE peaks, deleterious non-synonymous sites, other putative functional sites and eQTLs. Taking the enrichment analysis for deleterious non-synonymous sites as an example, the empirical p is the proportion of all SNPs which were annotated with probably damaging or possibly damaging by PolyPhen or deleterious by SIFT in the whole genome. Then, for n SNPs that are significant SNPs or their LD proxies, if m out of the n SNPs were annotated to be deleterious non-synonymous sites, then enrichment *P*-value will be calculated as $\sum_{i=0}^{m-1}\binom{n}{i}p^{i}(1-p)^{n-i}$. For ENCODE regions, *P*-values are corrected by Bonferroni correction for number of ENCODE tracks.

Supplementary Tables

Supplementary Table 1 Statistics of SNPs annotated with functional data from Ensembl.

**Annotation Type in *i*-GSEA4GWAS v2**	**No. of SNPs [a]**	**Annotation Type in Ensembl**	**No. of SNPs [a]**
		benign (PolyPhen)	601,237
		unknown (PolyPhen)	97,300
**deleterious non-synonymous**	**645,100**	probably damaging (PolyPhen)	346,603
		possibly damaging (PolyPhen)	284,233
		deleterious (SIFT)	478,287
		tolerated (SIFT)	650,648
**others [b]**	**276,363**	splice donor variant	18,033
		stop lost	2,020
		incomplete terminal codon variant	925
		inframe insertion	2,612
		transcript ablation	87
		splice acceptor variant	14,752
		frameshift variant	22,262
		stop gained	32,728
		initiator codon variant	3,729
		splice region variant	182,469
		inframe deletion	3,447
		missense variant	1,073,227

[a] No. of SNPs mapped to this type of annotation. Some SNPs may be annotated into more than one annotation type.
[b] Others include splice donor variant, stop lost, incomplete terminal codon variant, inframe insertion, transcript ablation, splice acceptor variant, frameshift variant, stop gained, initiator codon variant, splice region variant or inframe deletion.

Supplementary Table 2 Sources of ENCODE data used for SNP functional analysis.

**Peak Type**	**Description**	**No. of Tracks**
DNase-seq Peaks	DNase-seq Peaks of Open Chromatin	125
FAIRE Peaks	FAIRE Peaks of Open Chromatin	24
TFBS Peaks (SPP)	TFBS SPP-based Peaks	495
TFBS Peaks (PeakSeq)	TFBS PeakSeq-based Peaks	495
Histone Peaks	Histone Peaks (only regions marked by active-associated histones, including H3K4me1, H3K4me2, H3K4me3, H3K9ac, H3K27ac, H3K36me3, H3K79me2, H4K20me1 and H3K9me1, were included as rSNPBase (Guo et al., 2014))	190

Supplementary Table 3 Sources of eQTLs data for SNP functional analysis.

**DB Source**	**Tissue**	**Reference**	**Track/File Name**	**No. of Terms**
eQTL_Browser	Fibroblasts	(Dimas et al., 2009)	Dimas09_fibroQTL	527
eQTL_Browser	lymphoblastoid cell lines	(Dimas et al., 2009)	Dimas09_lymphoQTL	563
eQTL_Browser	T-cells	(Dimas et al., 2009)	Dimas09_TcellsQTL	547
eQTL_Browser	lymphoblastoid cell lines	(Gaffney et al., 2012)	Gaffney12_eQTL	1,894
eQTL_Browser	Liver	(Innocenti et al., 2011)	Innocenti2011_eQTL	1,983
eQTL_Browser	lymphoblastoid cell lines	(Mangravite et al., 2013)	Mangravite12_eQTL	62,666
eQTL_Browser	Cortex	(Myers et al., 2007)	Myers	769
eQTL_Browser	lymphoblastoid cell lines	(Pickrell et al., 2010)	Pickrell10_eQTL	984
eQTL_Browser	Liver	(Schadt et al., 2008)	Schadt	5,578
eQTL_Browser	lymphoblastoid cell lines	(Stranger et al., 2007)	Stranger	23,717
eQTL_Browser	lymphoblastoid cell lines	(Veyrieras et al., 2008)	Veyrieras_PP	8,577
eQTL_Browser	lymphoblastoid cell lines	(Veyrieras et al., 2008)	Veyrieras_Pvalue	16,550
eQTL_Browser	Monocytes	(Zeller et al., 2010)	Zeller10_QTL	53,935
GTEx	Brain Cerebellum	(Gibbs et al., 2010)	Brain_cerebellum.tab	5,243
GTEx	Brain Frontal Cortex	(Gibbs et al., 2010)	Brain_frontal_cortex.tab	5,512
GTEx	Brain Temporal Cortex	(Gibbs et al., 2010)	Brain_temporal_cortex.tab	5,335
GTEx	Brain Pons	(Gibbs et al., 2010)	Brain_pons.tab	3,411
GTEx	Lymphoblastoid	(Montgomery et al., 2010)	Lymphoblastoid.tab	5,364
seeQTL	HapMap human lymphoblastoid cell lines	(Xia et al., 2012)	Qvalue_cutoff_hapmap3_cis_hg19.txt	75,779
seeQTL	HapMap human lymphoblastoid cell lines	(Xia et al., 2012)	Qvalue_cutoff_hapmap3_trans_hg19.txt	51,369
**Total**				**330,303**
**After removed non-approved gene symbol**				**313,594**
**Unique SNPs**				**197,945**

Supplementary Table 4 The analysis result of *i*-GSEA4GWAS v2 for schizophrenia GWAS data.

Pathway Name	Pathway ID	*P*-value	FDR	*P*-value of enrichment analysis for putative functional variants		# Significantly enriched peaks (*P*-value<0.05)					*P*-value of enrichment analysis for eQTL
				Deleterious	Others[a]	DNase-seq	FAIRE	TFBS-PeakSeq	TFBS-SPP	Histone	
potassium ion transport	GO:0006813	0.001	0.002	0.956	0.8768	0	1	0	0	0	0.7766
antigen processing and presentation	hsa04612	0.001	0.004	0.9458	0.00868	0	0	0	0	0	1.98E-103
cation transport	GO:0006812	0.001	0.0042	0.999	0.8547	0	0	0	0	0	7.65E-48
monovalent inorganic cation transport	GO:0015672	0.001	0.0042	0.9815	0.9204	0	0	0	0	0	0.0367
ion channel activity	GO:0005216	0.001	0.0043	0.9986	1	0	0	0	0	0	0.00000095
ion transport	GO:0006811	0.001	0.0043	1	0.7844	0	0	0	0	0	7.75E-45
substrate specific channel activity	GO:0022838	0.001	0.0061	0.9987	1	0	0	0	0	0	0.00000108
metal ion transport	GO:0030001	0.001	0.0082	0.9972	0.6704	0	0	0	0	0	3.33E-27
gated channel activity	GO:0022836	0.001	0.0104	0.9971	1	0	0	0	0	0	0.0000501
regulation of heart contraction	GO:0008016	0.001	0.0112	1	1	0	1	0	0	0	0.0839
voltage gated potassium channel complex	GO:0008076	0.001	0.0327	0.8557	1	0	1	0	0	0	0.6492
auxiliary transport protein activity	GO:0015457	0.001	0.0417	1	1	2	0	0	0	0	0.000396
cell adhesion molecules cams	hsa04514	0.001	0.0432	0.9984	0.3164	0	0	1	8	1	3.39E-69
Endocytosis	hsa04144	0.002	0.0447	1	0.3726	0	0	0	0	0	2.17E-81
potassium channel activity	GO:0005267	0.001	0.0448	0.9222	1	0	1	0	0	0	0.7237
channel regulator activity	GO:0016247	0.001	0.0453	1	1	2	0	0	0	0	0.00017
activation of protein kinase activity	GO:0032147	0.002	0.0458	1	1	0	1	0	0	17	1.1E-44
protein processing	GO:0016485	0.002	0.0468	0.935	0.6886	0	0	0	0	0	0.052
nicotinate and nicotinamide metabolism	hsa00760	0.003	0.0471	1	1	0	0	0	0	0	5.31E-16

[a] Others include splice donor variant, stop lost, incomplete terminal codon variant, inframe insertion, transcript ablation, splice acceptor variant, frameshift variant, stop gained, initiator codon variant, splice region variant or inframe deletion.

Supplementary Table 5 Enriched DNase I hypersensitive sites and TFBS peaks by pathways identified from schizophrenia GWAS data.

Pathway/Gene set name	Enriched ENCODE Tracks					
	**Type**	**Track**	**Cell Line**	**Cell Type**	**Tissue**	***P*-value**
potassium ion transport	FAIRE	wgEncodeOpenChromFaireMedulloPk	Medullo	Medulloblastoma	brain	0.00147
regulation of heart contraction	FAIRE	wgEncodeOpenChromFaireMedulloPk	Medullo	Medulloblastoma	brain	5.65E-05
voltage gated potassium channel complex	FAIRE	wgEncodeOpenChromFaireMedulloPk	Medullo	Medulloblastoma	brain	5.86E-04
auxiliary transport protein activity	DNase-seq	wgEncodeDukeDnaseOsteobl	Osteobl	Osteoblast	bone	2.78E-04
	DNase-seq	wgEncodeDukeDnaseFibrobl	Fibrobl	Fibroblast	skin	2.83E-04
cell adhesion molecules cams	TFBS-PeakSeq	wgEncodeSydhTfbsK562Nrf1IggrabAlnRep0	K562	Leukemia cell	blood	0.0000297
	TFBS-SPP	wgEncodeSydhTfbsH1hescNrf1IggrabAlnRep0	H1-hESC	Embryonic stem cell	embryonic	3.07E-06
	TFBS-SPP	wgEncodeSydhTfbsK562Nrf1IggrabAlnRep0	K562	Leukemia cell	blood	3.73E-06
	TFBS-SPP	wgEncodeSydhTfbsHelas3E2f6StdAlnRep0	HeLa-S3		cervix	0.0000049
	TFBS-SPP	wgEncodeSydhTfbsHelas3E2f1StdAlnRep0	HeLa-S3		cervix	6.18E-06
	TFBS-SPP	wgEncodeHaibTfbsHepg2Hnf4gsc6558V0416101AlnRep0	HepG2	Carcinoma	liver	0.0000148
	TFBS-SPP	wgEncodeHaibTfbsHepg2Hnf4ah171Pcr1xAlnRep0	HepG2	Carcinoma	liver	0.0000249
	TFBS-SPP	wgEncodeSydhTfbsHelas3Elk4UcdAlnRep0	HeLa-S3		cervix	0.0000344
	TFBS-SPP	wgEncodeSydhTfbsHelas3Hae2f1StdAlnRep0	HeLa-S3		cervix	0.0000807
potassium channel activity	FAIRE	wgEncodeOpenChromFaireMedulloPk	Medullo	Medulloblastoma	brain	9.91E-04
channel regulator activity	DNase-seq	wgEncodeDukeDnaseOsteobl	Osteobl	Osteoblast	bone	2.58E-04
	DNase-seq	wgEncodeDukeDnaseFibrobl	Fibrobl	Fibroblast	skin	2.63E-04
activation of protein kinase activity	FAIRE	wgEncodeOpenChromFaireHelas3Ifna4hPk	HeLa-S3		cervix	0.000527

Supplementary Table 6 Comparison between *i*-GSEA4GWAS v2 and web-based tools (including databases) for functional analysis.

**Tool**	**LD-proxy**	**Coding features**	**No-coding features**	**eQTLs**	**Enrichment analysis**
i-GSEA4GWAS v2	Yes	Yes	Yes	Yes	Yes
GREAT (McLean et al., 2010)	No	No	Yes	No	Yes
GenomeRunner Web (Dozmorov et al., 2012)	No	No	Yes	No	Yes
ChroMos (Barenboim and Manke, 2013)	No	No	Yes	No	No
rSNPBase (Guo et al., 2014)	Yes	No	Yes	Yes	No
is-rSNP (Macintyre et al., 2010)	No	No	Yes	No	No
GWASrap (Li et al., 2012)	Yes	Yes	Yes	Yes	No
TRAP (Thomas-Chollier et al., 2011)	No	No	Yes	No	No
HaploReg (Ward and Kellis, 2012)	Yes	No	Yes	Yes	Yes
RegulomeDB (Boyle et al., 2012)	No	No	Yes	No	No
GWAS3D (Li et al., 2013)	Yes	No	Yes	No	Yes
GERP++ (Davydov et al., 2010)	No	No	Yes	No	No
dbPSHP (Li et al., 2014)	No	No	Yes	No	No

References

Abecasis, G.R., Auton, A., Brooks, L.D., DePristo, M.A., Durbin, R.M., Handsaker, R.E., Kang, H.M., Marth, G.T., and McVean, G.A. (2012). An integrated map of genetic variation from 1,092 human genomes. Nature 491, 56-65.

Altshuler, D.M., Gibbs, R.A., Peltonen, L., Dermitzakis, E., Schaffner, S.F., Yu, F., Bonnen, P.E., de Bakker, P.I., Deloukas, P., Gabriel, S.B., *et al.* (2010). Integrating common and rare genetic variation in diverse human populations. Nature 467, 52-58.

Barenboim, M., and Manke, T. (2013). ChroMoS: an integrated web tool for SNP classification, prioritization and functional interpretation. Bioinformatics 29, 2197-2198.

Boyle, A.P., Hong, E.L., Hariharan, M., Cheng, Y., Schaub, M.A., Kasowski, M., Karczewski, K.J., Park, J., Hitz, B.C., Weng, S., *et al.* (2012). Annotation of functional variation in personal genomes using RegulomeDB. Genome Res 22, 1790-1797.

Davydov, E.V., Goode, D.L., Sirota, M., Cooper, G.M., Sidow, A., and Batzoglou, S. (2010). Identifying a high fraction of the human genome to be under selective constraint using GERP++. PLoS Comput Biol 6, e1001025.

Dimas, A.S., Deutsch, S., Stranger, B.E., Montgomery, S.B., Borel, C., Attar-Cohen, H., Ingle, C., Beazley, C., Gutierrez Arcelus, M., Sekowska, M., *et al.* (2009). Common regulatory variation impacts gene expression in a cell type-dependent manner. Science 325, 1246-1250.

Dozmorov, M.G., Cara, L.R., Giles, C.B., and Wren, J.D. (2012). GenomeRunner: automating genome exploration. Bioinformatics 28, 419-420.

Gaffney, D.J., Veyrieras, J.B., Degner, J.F., Pique-Regi, R., Pai, A.A., Crawford, G.E., Stephens, M., Gilad, Y., and Pritchard, J.K. (2012). Dissecting the regulatory architecture of gene expression QTLs. Genome Biol 13, R7.

Gibbs, J.R., van der Brug, M.P., Hernandez, D.G., Traynor, B.J., Nalls, M.A., Lai, S.L., Arepalli, S., Dillman, A., Rafferty, I.P., Troncoso, J., *et al.* (2010). Abundant quantitative trait loci exist for DNA methylation and gene expression in human brain. PLoS Genet 6, e1000952.

Guo, L., Du, Y., Chang, S., Zhang, K., and Wang, J. (2014). rSNPBase: a database for curated regulatory SNPs. Nucleic Acids Res 42, D1033-1039.

Innocenti, F., Cooper, G.M., Stanaway, I.B., Gamazon, E.R., Smith, J.D., Mirkov, S., Ramirez, J., Liu, W., Lin, Y.S., Moloney, C., *et al.* (2011). Identification, replication, and functional fine-mapping of expression quantitative trait loci in primary human liver tissue. PLoS Genet 7, e1002078.

Li, M.J., Sham, P.C., and Wang, J. (2012). Genetic variant representation, annotation and prioritization in the post-GWAS era. Cell Res 22, 1505-1508.

Li, M.J., Wang, L.Y., Xia, Z., Sham, P.C., and Wang, J. (2013). GWAS3D: Detecting human regulatory variants by integrative analysis of genome-wide associations, chromosome interactions and histone modifications. Nucleic Acids Res 41, W150-158.

Li, M.J., Wang, L.Y., Xia, Z., Wong, M.P., Sham, P.C., and Wang, J. (2014). dbPSHP: a database of recent positive selection across human populations. Nucleic Acids Res 42, D910-916.

Macintyre, G., Bailey, J., Haviv, I., and Kowalczyk, A. (2010). is-rSNP: a novel technique for in silico regulatory SNP detection. Bioinformatics 26, i524-530.

Mangravite, L.M., Engelhardt, B.E., Medina, M.W., Smith, J.D., Brown, C.D., Chasman, D.I., Mecham, B.H., Howie, B., Shim, H., Naidoo, D., *et al.* (2013). A statin-dependent QTL for GATM expression is associated with statin-induced myopathy. Nature 502, 377-380.

McLean, C.Y., Bristor, D., Hiller, M., Clarke, S.L., Schaar, B.T., Lowe, C.B., Wenger, A.M., and Bejerano, G. (2010). GREAT improves functional interpretation of cis-regulatory regions. Nat Biotechnol 28, 495-501.

Montgomery, S.B., Sammeth, M., Gutierrez-Arcelus, M., Lach, R.P., Ingle, C., Nisbett, J., Guigo, R., and Dermitzakis, E.T. (2010). Transcriptome genetics using second generation sequencing in a Caucasian population. Nature 464, 773-777.

Myers, A.J., Gibbs, J.R., Webster, J.A., Rohrer, K., Zhao, A., Marlowe, L., Kaleem, M., Leung, D., Bryden, L., Nath, P., *et al.* (2007). A survey of genetic human cortical gene expression. Nat Genet 39, 1494-1499.

Pickrell, J.K., Marioni, J.C., Pai, A.A., Degner, J.F., Engelhardt, B.E., Nkadori, E., Veyrieras, J.B., Stephens, M., Gilad, Y., and Pritchard, J.K. (2010). Understanding mechanisms underlying human gene expression variation with RNA sequencing. Nature 464, 768-772.

Schadt, E.E., Molony, C., Chudin, E., Hao, K., Yang, X., Lum, P.Y., Kasarskis, A., Zhang, B., Wang, S., Suver, C., *et al.* (2008). Mapping the genetic architecture of gene expression in human liver. PLoS Biol 6, e107.

Stranger, B.E., Nica, A.C., Forrest, M.S., Dimas, A., Bird, C.P., Beazley, C., Ingle, C.E., Dunning, M., Flicek, P., Koller, D., *et al.* (2007). Population genomics of human gene expression. Nat Genet 39, 1217-1224.

Thomas-Chollier, M., Hufton, A., Heinig, M., O'Keeffe, S., Masri, N.E., Roider, H.G., Manke, T., and Vingron, M. (2011). Transcription factor binding predictions using TRAP for the analysis of ChIP-seq data and regulatory SNPs. Nat Protoc 6, 1860-1869.

Veyrieras, J.B., Kudaravalli, S., Kim, S.Y., Dermitzakis, E.T., Gilad, Y., Stephens, M., and Pritchard, J.K. (2008). High-resolution mapping of expression-QTLs yields insight into human gene regulation. PLoS Genet 4, e1000214.

Ward, L.D., and Kellis, M. (2012). HaploReg: a resource for exploring chromatin states, conservation, and regulatory motif alterations within sets of genetically linked variants. Nucleic Acids Res 40, D930-934.

Xia, K., Shabalin, A.A., Huang, S., Madar, V., Zhou, Y.H., Wang, W., Zou, F., Sun, W., Sullivan, P.F., and Wright, F.A. (2012). seeQTL: a searchable database for human eQTLs. Bioinformatics 28, 451-452.

Zeller, T., Wild, P., Szymczak, S., Rotival, M., Schillert, A., Castagne, R., Maouche, S., Germain, M., Lackner, K., Rossmann, H., *et al.* (2010). Genetics and beyond--the transcriptome of human monocytes and disease susceptibility. PLoS One 5, e10693.

Zhang, K., Cui, S., Chang, S., Zhang, L., and Wang, J. (2010). i-GSEA4GWAS: a web server for identification of pathways/gene sets associated with traits by applying an improved gene set enrichment analysis to genome-wide association study. Nucleic Acids Res 38, W90-95.
